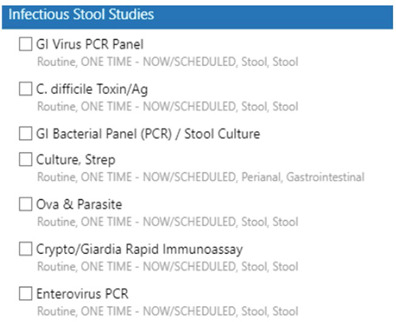# The Power of Suggestion: Irrelevant Test Options in Order Panels, an Interrupted Time Series with Cost Analysis

**DOI:** 10.1017/ash.2025.316

**Published:** 2025-09-24

**Authors:** Nathan L’Etoile, Yun Li, Sanjeev Swami, Tracey Polsky, Kenneth Smith

**Affiliations:** 1 Children’s Hospital of Philadelphia

## Abstract

**Introduction:** Grouping of medical tests in an order panel or set may facilitate standardized care but could have the unintended consequence of increasing unnecessary testing. At our institution, one such panel includes studies performed on stool for the purposes of diagnosing infectious diarrhea (Figure 1). We removed stool enterovirus polymerase chain reaction (PCR) from this order panel given limited data supporting its use in the diagnosis of the etiology of diarrhea. **Objectives:** We aimed to evaluate the impact of removing the stool enterovirus PCR from this panel and whether there were associated decreased costs from this intervention. **Methods:** We conducted an interrupted time series to estimate the initial impact of implementing this order panel, followed by the later removal of the enterovirus order from the panel, using gastrointestinal (GI) bacterial PCR orders as a control. Additionally, we conducted a cost-savings analysis by multiplying the cost per test by the decrease in tests/month after removing the order from this panel averaged over a year. **Results:** After the panel’s creation, there was an immediate significant increase in enterovirus stool PCR ordering from a predicted mean of 28 tests/month to 43 tests/month (difference of 15 tests/month, p < 0 .0001) (Figure 2, blue). Similarly, the bacterial stool PCR ordering increased from a predicted mean of 98 tests/month to 136 tests/month (increased by 37 in the month following panel creation, p < 0 .0001). Conversely, after the removal of enterovirus PCR from the panel, there was an immediate significant decrease in testing from a predicted mean of 60 tests/month to 17 tests/month (decreased by 43 tests/month, p < 0 .0001), without a significant change in bacterial stool PCR ordering (16 test/month decrease, p=0.10) (Figure 2, red). We estimate that this simple intervention will save an average of $8,500 annually in direct costs each year. **Discussion:** Enterovirus PCR ordering significantly increased after the introduction of an order panel bundling stool studies targeted at diagnosing diarrhea. When this order was removed from the panel, there was a significant decrease in ordering without a change in infectious stool testing overall, as evidenced by no significant change in GI bacterial panel ordering. We hypothesize that clinicians utilize this panel to craft a differential for acute-onset diarrhea. Therefore, when the stool enterovirus PCR option was removed from this panel, it is possible that it was no longer considered on the differential. Reviewing such order panels may be helpful in reducing unnecessary testing and costs to healthcare systems.

**Figure:**
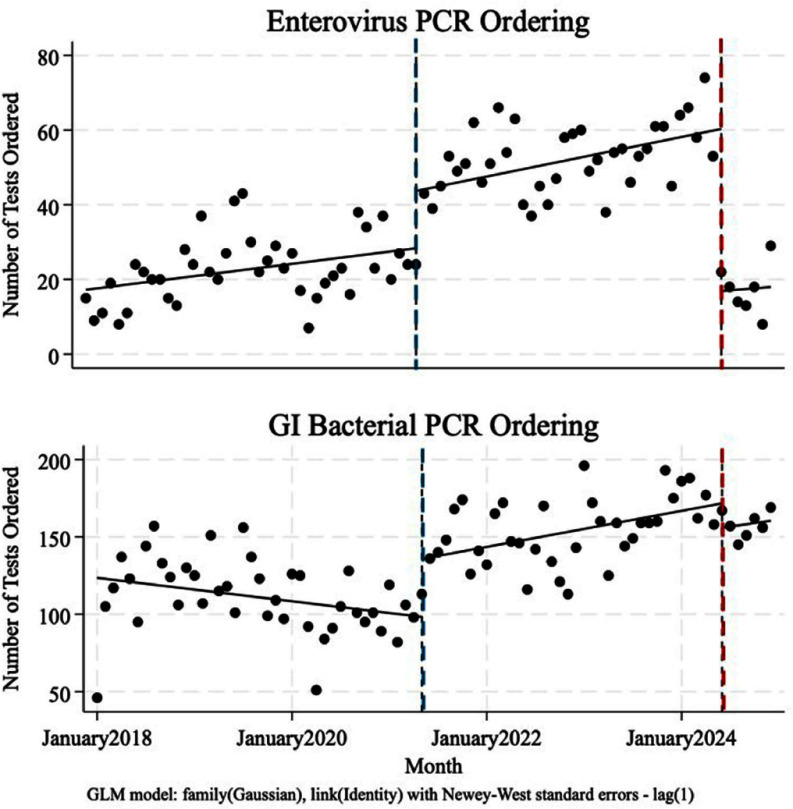

**Figure:**